# The Wheat Endophyte *Epicoccum layuense* J4-3 Inhibits *Fusarium graminearum* and Enhances Plant Growth

**DOI:** 10.3390/jof10010010

**Published:** 2023-12-24

**Authors:** Clement Nzabanita, Lihang Zhang, Yanfei Wang, Shuangchao Wang, Lihua Guo

**Affiliations:** State Key Laboratory for Biology of Plant Diseases and Insect Pests, Institute of Plant Protection, Chinese Academy of Agricultural Sciences, Beijing 100193, China; nclement024@gmail.com (C.N.); lhzhang0911@163.com (L.Z.); 15735648812@163.com (Y.W.); wangshuangchao@caas.cn (S.W.)

**Keywords:** biocontrol agent, *Epicoccum layuense*, *Fusarium graminearum*, Fusarium head blight, plant growth promotion, wheat

## Abstract

Fungal endophytes are well-known for their ability to promote plant growth and hinder fungal diseases, including Fusarium head blight (FHB) caused by *Fusarium graminearum*. This study aimed to characterize the biocontrol efficacy of strain J4-3 isolated from the stem of symptomless wheat collected from Heilongjiang Province, China. It was identified as *Epicoccum layuense* using morphological characteristics and phylogenetic analysis of the rDNA internal transcribed spacer (ITS) and beta-tubulin (*TUB*). In a dual culture assay, strain J4-3 significantly inhibited the mycelial growth of *F. graminearum* strain PH-1 and other fungal pathogens. In addition, wheat coleoptile tests showed that lesion symptoms caused by *F. graminearum* were significantly reduced in wheat seedlings treated with hyphal fragment suspensions of strain J4-3 compared to the controls. Under field conditions, applying spore suspensions and culture filtrates of strain J4-3 with conidial suspensions of *F. graminearum* on wheat spikes resulted in the significant biocontrol efficacy of FHB. In addition, wheat seedlings previously treated with spore suspensions of strain J4-3 before sowing successfully resulted in FHB reduction after the application of conidial suspensions of *F. graminearum* at anthesis. More importantly, wheat seedlings treated with hyphal fragments and spore suspensions of strain J4-3 showed significant increases in wheat growth compared to the controls under greenhouse and field conditions. Overall, these findings suggest that *E. layuense* J4-3 could be a promising biocontrol agent (BCA) against *F. graminearum*, causing FHB and a growth-promoting fungus in wheat.

## 1. Introduction

Wheat (*Triticum aestivum* L.) is a key food grain source that provides minerals, vitamins, essential amino acids, and fiber and serves as a staple food for humans and livestock [1,2]. Thus, it is of utmost concern to steadily increase wheat yield while minimizing potential losses [3]. Wheat, like other plants, is attacked by various fungal pathogens. *Fusarium graminearum* is one of the major pathogens causing disease in wheat and can affect all its growth stages [4]. *F. graminearum* is associated with severe disease, FHB, causing notable reductions in grain quality and quantity after infection not only in wheat but also in other cereal crops, including maize (*Zea mays* L.), barley (*Hordeum vulgare* L.), triticale (×*Triticosecale* Wittmack), and oats (*Avena sativa* L.) [5,6,7].

In addition to quality and quantity losses, disease progress is accompanied by the production of mycotoxins, including zearalenone (ZEN), nivalenol (NIV), and deoxynivalenol (DON), that are carcinogenic to the health of humans and animals [8,9,10,11,12]. Therefore, being one of the most destructive diseases of wheat, FHB is regarded as one of the most damaging factors in cereal production and attracts the attention of plant pathologists worldwide [13].

Currently, because of the limitations of highly resistant wheat cultivars, chemical fungicides are the most effective process of controlling FHB [14]. Nonetheless, non-selective use of commercial fungicides at specific concentrations can induce mycotoxin biosynthesis and result in the appearance of *F. graminearum* populations resistant to chemical fungicides [10]. In addition, the overuse of chemical fungicides has resulted in severe ecological problems, such as ecosystem destruction, increased resistance of target pests, and pollution of the environment [3,15,16,17]. Therefore, to avoid this predicament, various management strategies with eco-friendly, effective, and different modes of action need to be studied [10].

Biological controls, for instance, include the use of fungal endophytes that produce bioactives to reduce the invasion of plant pathogens and are one of the new alternatives to control plant fungal diseases, as they are safer, environmentally friendly, and supportable alternatives to chemical agents [18,19]. In addition, the use of BCAs in agriculture is increasingly widespread due to their long-lasting mechanisms for controlling fungal pathogens and enhancing plant growth [20]. The BCAs produce antagonistic secondary metabolites that primarily aim to inhibit fungal conidial germination, damaging membranes and cell walls, interacting with a variety of intracellular targets, leaking macromolecular substances, and accumulating endogenous active substances in mycelial cells to damage fungal pathogens [21].

Fungal endophytes are latent BCAs against various fungal pathogens and promote the growth and health of plants. These fungi are mutualistically associated with their hosts and do not cause any visible disease symptoms [22]. The benefit of using fungal endophytes as BCAs is that they can adapt to live inside the plants and produce antagonistic secondary metabolites that are essential for their host plant’s growth (biofertilizer) and disease resistance [1,23] and increase plant growth and yields [24]. Indeed, secondary metabolites produced by fungal endophytes play an important role in virulence reduction through the production of antifungal and antimicrobial inhibitory compounds, and they may be linked to the non-symptoms observed in host plants [25,26]. 

There has been an increase in research on the ability of numerous genera of fungi to colonize the internal tissues of a large number of host plants [27,28,29] and subsequently confer numerous benefits, including suppression of pathogen diseases [30,31,32] and enhancement of plant growth [33,34,35]. Importantly, enhanced plant growth and reduced disease severity mediated by colonization of endophytes with diverse genera of fungal endophytes have been established following the fungal inoculation using different methods, including seed coating, foliar spray, and root drench, on wheat and other cereals [36,37]. Cotton seed inoculation with *Beauveria bassiana* and *Purpureocillium lilacinum* had significant growth-promoting effects on plant dry biomass, number of nodes, and reproductive tissues [35]. Similarly, treating broad bean seeds with *B. bassiana* and *Metarhizium brunneum* notably increased plant height, leaf pair number, and fresh shoot and root weights [33]. Additionally, wheat seeds treated with the fungal endophyte *S. sclerotiorum* not only provided protection against FHB and wheat rust but also enhanced the growth and yield of wheat [38]. 

Under biotic and abiotic stresses, the fungal endophytes from the genera *Chaetomium*, *Curvularia*, *Epicoccum*, *Penicillium*, *Serentipita*, and *Trichoderma* resulted in the control of fungal diseases and enhanced plant growth and yield [39,40]. Importantly, endophytes belonging to the genus *Epicoccum* are well-known as BCAs against numerous phytopathogenic fungi [20,41,42].

However, despite extensive research on the bioactivity of *Epicoccum* spp., studies on *E. layuense* are still not well documented. Therefore, the objective of this study was to elucidate the biocontrol potential of *E. layuense* strain J4-3 against FHB caused by *F. graminearum* and its growth-promoting efficiency in wheat. Our findings revealed that the tested fungus may provide insights into biocontrol activities for future applications.

## 2. Materials and Methods

### 2.1. Pathogenic Fungal Strains and Culture Conditions

Fungal pathogens *Fusarium graminearum* strain PH-1, *Botrytis cinerea*, *Colletotrichum gloeosporioides*, and *Sclerotinia sclerotiorum* were obtained from the fungal culture collection of the State Key Laboratory for Biology of Plant Diseases and Insect Pests, Institute of Plant Protection, Chinese Academy of Agricultural Sciences, Beijing, China. They were cultured on potato dextrose agar (PDA) at 25 °C in the darkness and securely stored in 25% glycerol at a temperature of −80 °C for long-term storage.

### 2.2. Endophyte Isolation and Morphological Characterization 

The isolation method was performed following Cao et al. [43] and Xu et al. [44] with slight modifications. Briefly, stems of symptomless winter wheat (cv. Longmai35) collected at the flowering stage from Heilongjiang Province, China (50°23′57″ N and 127°57′75″ E), were thoroughly washed in tap water, and each stem was cut into small segments in length using sterile scalpels. The segments were surface sterilized by soaking with 70% alcohol for 30 s and 2% sodium hypochlorite for 1 min. They were then washed three times in sterile distilled water and dried on sterile filter paper. Tissues (~3–5 mm diameter) were placed into potato dextrose agar (PDA) plates supplemented with ampicillin (50 µg/mL) and kanamycin (50 µg/mL) to inhibit the growth of bacteria. To assess the efficacy of the surface sterilization, aliquots (100 µL) from the final washed water were also plated on the PDA plates. Further, the plates were incubated at 25 °C in darkness for 3–4 days. Pure colonies were obtained by repeatedly transferring 1–2 mm hyphal tips onto fresh PDA plates. 

Strain J4-3, showing similarities to *Epicoccum* spp., was subjected to a detailed examination. To do so, the colony morphologies of strain J4-3 were analyzed after incubation at 25 °C for 7–14 days on PDA plates. In addition, conidia were prepared by culturing its mycelial plugs into potato sucrose agar (PSA; potato 200 g/L, sucrose 20 g/L, and agar 16 g/L) for 14 days and harvested according to Cao et al. [43]. The conidia characteristics were observed using a bright-field microscope (Leica CTR6, Leica Biosystems, Wetzlar, Germany). In addition, the effect of pH on the growth of strain J4-3 was further determined by removing its mycelial plugs (5 mm) from the colony edges and transferring them into fresh PDA plates with the pH previously adjusted to 5.0, 6.0, 7.0, 8.0, 9.0, and 10.0 through the addition of 0.1 M NaOH or HCl before sterilization. The plates were cultured at 25 °C under dark conditions, and the colony diameter was measured weekly for 3 weeks. At each pH, the growth rate was calculated by dividing the colony diameter by the number of days.

### 2.3. DNA Extraction, PCR Amplification, Sequencing, and Phylogenetic Analysis

Genomic DNA was extracted from mycelia grown on PDA overlaid with a cellophane membrane using the cetyltrimethylammonium bromide (CTAB)-based method [45]. The NanoDrop 2000 spectrophotometer (Thermo Scientific, Waltham, MA, USA) was used to measure the DNA concentration. The ITS was amplified with the primer pairs ITS1 and ITS4 [46], and *TUB* was amplified with the primers Btub2Fd and Btub4Rd [47]. The PCR amplifications were carried out in a T100 thermal cycler (Bio-Rad) in a total volume of 25 μL containing genomic DNA 1 μL, each of the forward and reverse primers 1 μL, Green Taq Mix (Vazyme, Beijing, China) 12.5 μL, and double-deionized water (ddH_2_O) 9.5 μL. The PCR conditions for ITS and *TUB* were performed as follows: an initial denaturation step at 94 °C for 5 min followed by 35 cycles at 94 °C for 30 s, 55 °C for 30 s, 72 °C for 1 min of annealing, and a final extension at 72 °C for 10 min. The PCR products were visualized after electrophoresis on 1% agarose gel, then purified and sequenced bidirectionally by Tsingke Biotech Co., Ltd. (Beijing, China). SeqMan software v7.1 (DNASTAR Lasergene, Madison, WI, USA) and the BLAST program on the NCBI website (http://blast.st-va.ncbi.nlm.nih.gov/Blast.cgi) were used to assemble and analyze the sequencing data, respectively. The sequences of ITS and *TUB* from strain J4-3 and the reference sequence of *Epicoccum* downloaded from GenBank were selected for phylogenetic analyses. Maximum-likelihood phylogenetic trees were individually constructed for each DNA locus using MEGA version 7.0. 

### 2.4. In Vitro Dual Culture Activity of Strain J4-3

The assessment of the inhibitory effects of strain J4-3 against the mycelial growth of the aforementioned fungal pathogens was carried out using the dual-culture method [48]. In brief, mycelial discs (5 mm) of fungal pathogens taken from 7-day-old cultures were inoculated at the center of PDA contained in 90 mm diameter Petri plates. Then, the same-sized mycelial discs of strain J4-3 were placed at equal distances from the periphery of the Petri plate on the same diagonal line. Plates inoculated only with pathogens served as controls. Plates were incubated in the dark at 25 °C for 7 days. After the incubation period, the mycelial radial growth (mm) and the size of the inhibition zone were measured, and the percent inhibition of radial growth (PIRG) was determined using the formula:

PIRG = [(Rc − Rt)/Rc] × 100

where Rc = mycelial radial growth of pathogens in the control and Rt = mycelial radial growth of pathogens in dual culture experiments with the antagonist. 

### 2.5. Effect of Hyphal Fragment Suspensions of J4-3 against F. graminearum by Wheat Coleoptile Assay

To examine whether wheat seedlings treated with hyphal fragment suspensions of J4-3 could inhibit *F. graminearum* infection, a wheat coleoptile assay was conducted in a greenhouse at 25 °C, 65% relative humidity (RH), with 16 h/8 h photoperiod, using winter wheat (cv. Jimai22). The hyphal fragment suspensions of strain J4-3 were obtained according to Yu et al. [49], with minor modifications. In brief, fresh mycelia of 5-day-old cultures were collected from PDA medium, and then small mycelial plugs were cultured in potato dextrose broth (PDB) medium in 250-mL flasks in a shaker at 25 °C for 7 days at 175 rpm. Fungal fragments were broken apart using a sterile blender and adjusted to an OD_600_ = 1.0. Meanwhile, conidia of *F. graminearum* were produced in carboxymethylcellulose (CMC) broth following Li et al. [50]. The conidial concentration was assessed using a hemocytometer and adjusted to 1 × 10^6^ conidia mL^−1^, and then immediately used to inoculate the wheat coleoptile. To do so, wheat seeds were surface sterilized with 5% NaClO for 5 min, then washed three times with sterile distilled water. They were cultured into Petri dishes containing sterile filter papers wetted with hyphal fragment suspensions of strain J4-3 for 4 days. After seed germination, seedlings were transferred into new Petri dishes containing sterile filter papers wetted with sterile distilled water. Additionally, the top 2 to 3 mm of the coleoptiles were removed, and the seedlings were covered with sterile cotton strips followed by inoculation with 20 μL of conidial suspension of *F. graminearum*. Mock-inoculated wheat coleoptiles treated with sterile distilled water served as controls. Subsequently, lesion sizes on the coleoptiles were measured at 7 dpi for statistical analysis. 

### 2.6. Biocontrol Activity of Spore Suspensions and Culture Filtrates of J4-3 against FHB in Field Conditions

To verify the biocontrol efficacy of J4-3 against FHB under field conditions, wheat seeds cv. Jimai22 (susceptible to FHB) were grown in the experimental field of the Institute of Plant Protection, Chinese Academy of Agricultural Sciences, Beijing, China (40°02′70″ N and 116°28′02″ E), until anthesis. Spores of strain J4-3 were prepared as mentioned above, and culture filtrates were obtained following Nzabanita et al. [48]. Two inoculation methods were performed as previously described with modifications [51]. (1) The point-inoculated method: a mixture (10 µL) of conidial suspensions of *F. graminearum* (1 × 10^6^ conidia mL^−1^) and spore suspensions (1 × 10^6^ spores mL^−1^) or culture filtrates of *E. layuense* at a ratio of 1:1 (*v*/*v*) were inoculated on individual spikelets. Inoculation of conidial suspensions of *F. graminearum* alone served as controls. (2) The spray-point inoculated method: approximately 5 mL of spore suspensions (1 × 10^6^ spores mL^−1^) or culture filtrates of *E. layuense* were applied on separate heads by spraying with a hand-held atomizer. At 1, 2, and 3 days post spraying, each treated wheat spike was inoculated with 10 μL of conidial suspension of *F. graminearum* (1 × 10^6^ conidia mL^−1^), respectively. Sterile distilled water sprayed instead of spore suspensions or culture filtrates of *E. layuense* served as controls. Before head inoculation, all treatments were combined with 0.01% *v*/*v* Tween 20. Subsequently, the third spikelets from the bottom were individually injected using a micropipette.

To further investigate whether wheat seedlings previously treated with spore suspensions of *E. layuense* before sowing could resist FHB, a field experiment was conducted at Shangzhuang experimental field, Beijing, China (40°06′25″ N and 116°22′26″ E). In brief, roots of germinated wheat (4-day-old seedlings) were soaked in spore suspensions of *E. layuense* (1 × 10^6^ spores mL^−1^) for 12 h. Seedlings treated with ddH_2_O served as controls. At the early flowering stage, 10 µL conidial suspensions (1 × 10^6^ conidia mL^−1^) of *F. graminearum* were used to individually inoculate the third spikelets. To maintain humidity, wheat heads were wrapped in plastic bags for the first two days. Inoculated wheat heads were collected, and spikes were imaged after 15 days of inoculation. The FHB infection rate and protection level were calculated for statistical analysis. 

### 2.7. Evaluating the Growth of Wheat Treated with Hyphal Fragment Suspensions of J4-3 in a Greenhouse

To determine whether strain J4-3 could promote the growth of wheat in a greenhouse, wheat seeds (cv. Jimai22) were washed with tap water and surface sterilized, as mentioned above. Surface-sterilized seeds were cultured into Petri dishes containing sterile filter papers wetted with ddH_2_O, and they were incubated at 25 °C for 4 days until germination. The germinated wheat seedlings were treated with hyphal fragment suspensions of strain J4-3 at OD_600_ = 2.0, using the root injuring method as follows: sprouted seedlings were removed from Petri dishes, the root tips (~2 mm) were cut off, and the roots were immersed in the hyphal fragment suspensions of strain J4-3 for 6 h. Seedlings soaked only in PDB served as controls. The inoculated seedlings (one seedling per pot) were planted in a greenhouse at 25 °C, 65% RH, with 16 h/8 h photoperiod using soil that was sterilized twice for 1 h at 121 °C, with a 24 h interval between autoclaves. Following 45 days of treatment, wheat growth parameters were measured, and data were used for statistical analysis. 

### 2.8. Evaluating the Growth of Wheat Treated with Spore Suspensions of J4-3 under Field Conditions

To further evaluate whether applying spore suspensions of J4-3 on wheat seedlings could increase wheat growth, surface sterilized wheat seeds (cv. Jimai 22) were allowed to germinate following the methods described above. Then, the germinated seedlings were soaked in spore suspensions (1 × 10^6^ conidia mL^−1^) of J4-3 for 12 h. Seedlings soaked only in ddH_2_O served as controls. Seedlings were grown at Shangzhuang experimental field, Beijing, China (40°06′25″ N and 116°22′26″ E). Field management was carried out following standard farmer practice, with the exception that no fungicide was used. At the flowering stage, plant shoot height, tiller number, spike length, and the number of spikelets per spike were measured. In addition, the weight of 1000 seeds was calculated after harvest. Measurement data for each group were calculated for statistical analysis.

### 2.9. Statistical Analysis

Data are expressed as mean ± standard error (s.e). Statistical analyses were performed using IBM SPSS statistics v. 20.0 (SPSS Inc., Chicago, IL, USA). Student’s *t*-tests and One-way ANOVA, followed by Tukey’s HSD post hoc tests, were used to determine the significant differences between two treatments and among different treatments, respectively. * *p* < 0.05 was considered statistically significant. GraphPad Prism version 9.0 was used to conduct graphics. The sample sizes (n) and biological replications for each statistical analysis are described in the figure legends.

## 3. Results

### 3.1. Morphological Identification of E. layuense Strain J4-3

**Figure 1:**
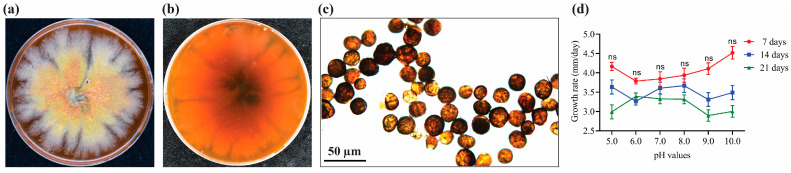
Morphological features of *E. layuense* strain J4-3. Colony morphology on PDA, (**a**,**b**) top and bottom views, (**c**) conidia. ImageJ software version 1.8.0 was used to measure the conidial length. (**d**) Growth rates (mm/day) of *E. layuense* strain J4-3 at varying pH after 7, 14, and 21 days of inoculation, respectively. At each pH, the growth rate was calculated by dividing the colony diameter by the number of days. Values are means ± s.e of six replicates. The experiment was repeated three times with similar results. ns indicates no significant difference between treatments at *p* < 0.05 level by Tukey’s HSD.

After 7 days of incubation at 25 °C on PDA, colonies of strain J4-3 were 52.7 ± 1.45 mm in diameter with yellow and even margins and floccose aerial mycelia; an orange-red diffusible pigment was evident on the PDA plate. At 14 days post-incubation, colonies were 68.9 ±1.8 mm in diameter and had irregular margins and aerial mycelia that were felty to floccose, flat, and buff to pink in color. Numerous conidia were produced on potato sucrose agar (PSA) and were globose to pyriform, turning to dark brown, and multicellular, measuring 15 to 23 μm, 19.12 ± 0.57 μm (*n* = 30) in diameter (Figure 1a–c). In addition, the pH results indicated that the strain J4-3 could grow on PDA at pH values of 5.0–10.0. Notably, there was no significant difference in the growth rate of strain J4-3 from pH 5.0 to 10.0 (Figure 1d).

### 3.2. Phylogenetic Analysis

**Figure 2:**
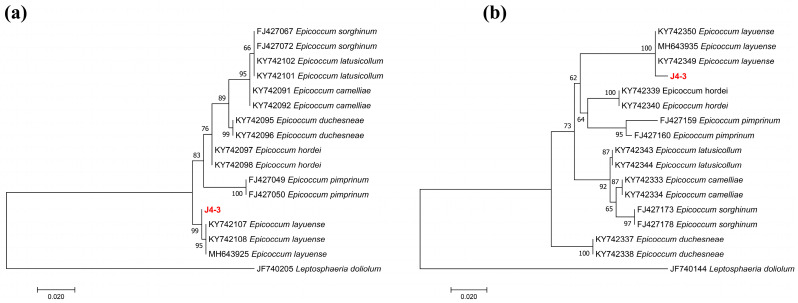
Phylogenetic tree of fungal endophyte *E. layuense* J4-3. (**a**,**b**) Individual maximum-likelihood trees for *E. layuense* strain J4-3 and related species based on the sequence data for ITS and *TUB*, respectively. *E. layuense* strain J4-3 is marked in bold red. Bootstrap values for maximum likelihood greater than 60% (1000 replicates) are given at the nodes. The scale bar represents nucleotide substitutions per site. *Leptosphaeria doliolum* CBS 505.75 was used as an outgroup taxon.

In individual maximum likelihood analyses of the ITS and *TUB*, strain J4-3 clustered with *E. layuense* (LC8155, LC8156, and E33) with 99% and 100% bootstrap support, respectively (Figure 2a,b). The ITS and *TUB* sequences of strain J4-3 were deposited in GenBank under accession numbers OR454088 and OR536426, respectively. The accession numbers from GenBank for the DNA loci analyzed in this study are summarized in Table S1. The tree was rooted to *Leptosphaeria doliolum* (CBS 505.75).

### 3.3. E. layuense Exhibits Broad-Spectrum Antifungal Activity

**Figure 3:**
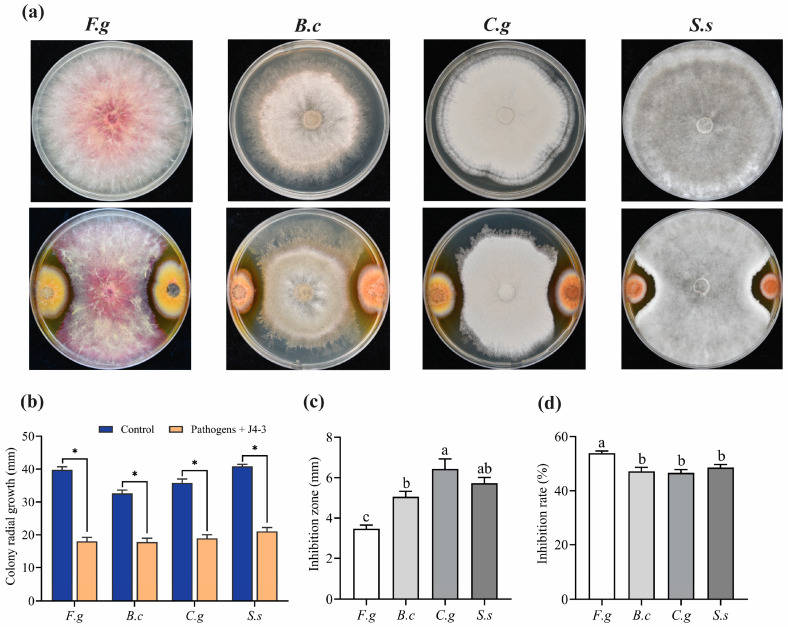
The broad-spectrum inhibition activity of *E. layuense* strain J4-3 against fungal pathogens. (**a**) Antifungal activity against phytopathogenic fungi using dual culture assay on PDA medium (*n* = 6). (**b**–**d**) The colony radial growth, inhibition zone, and inhibition rate (%) of fungal colony growth according to dual culture assay, respectively. Error bars represent standard error. Significant differences at *p* ≤ 0.05 are denoted as * according to Student’s *t*-tests or different lowercase letters using One-way ANOVA followed by Tukey’s HSD. The experiment was conducted in triplicate with similar results. *F. g*, *F. graminearum*; *B. c*, *B. cinerea*; *C. g*, *C. gloeosporioides*; and *S. s*, *S. sclerotiorum*.

It is widely known that numerous fungal plant pathogens are responsible for severe plant fungal diseases. Herein, dual culture tests showed that strain J4-3 had an inhibitory effect on fungal pathogens at different degrees compared to the controls (Figure 3a). The colony radial growth (mm) and inhibition zone produced by the fungal endophyte in front of the pathogen colonies were recorded (Figure 3b,c). In addition, the inhibition rates (%) against *F. g*, *B. c*, *C. g*, and *S. s* were 53.8%, 47.1%, 46.5%, and 48.5%, respectively (Figure 3d).

### 3.4. E. layuense Reduces F. graminearum Infection by Wheat Coleoptile Assay

**Figure 4:**
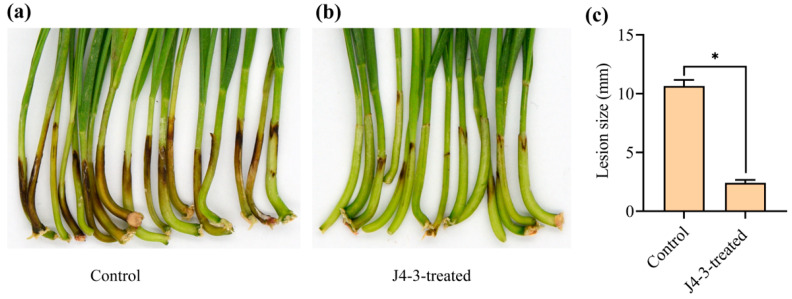
*E. layuense* reduces *F. graminearum* infection by wheat coleoptile assay. (**a**,**b**) Representative images of lesions on wheat seedlings infected by *F. graminearum* for the untreated (**a**) or treated seedlings (**b**) with hyphal fragment suspensions (OD_600_ = 1.0) of *E. layuense* strain J4-3. (**c**) Lesion lengths on wheat hypocotyls were measured at 7 days post-inoculation. Wheat coleoptiles were inoculated with 20 μL conidial suspensions (1 × 10^6^ conidia mL^−1^) of *F. graminearum*. Twenty wheat coleoptiles were used, and the experiment was repeated three times. Data are mean ± s.e. Significant differences at *p* ≤ 0.05 are denoted as * according to Student’s *t*-test.

In this study, the results revealed that seedlings treated with *E. layuense* strain J4-3 displayed shorter lengths and patches of dark brown lesions compared to the untreated controls (Figure 4a,b). The average length of lesions on the wheat coleoptiles infected with *F. graminearum* (control) was 10.66 ± 0.5 mm (*p* ≤ 0.05), whereas seedlings infected with *F. graminearum* treated with *E. layuense* showed an average lesion length of 2.42 ± 0.24 mm (*p* ≤ 0.05) (Figure 4c). Taken together, these results revealed that *E. layuense* plays an important role in the pathogenesis reduction of *F. graminearum* on wheat plants.

### 3.5. E. layuense Alleviates the Severity of FHB under Field Conditions

**Figure 5:**
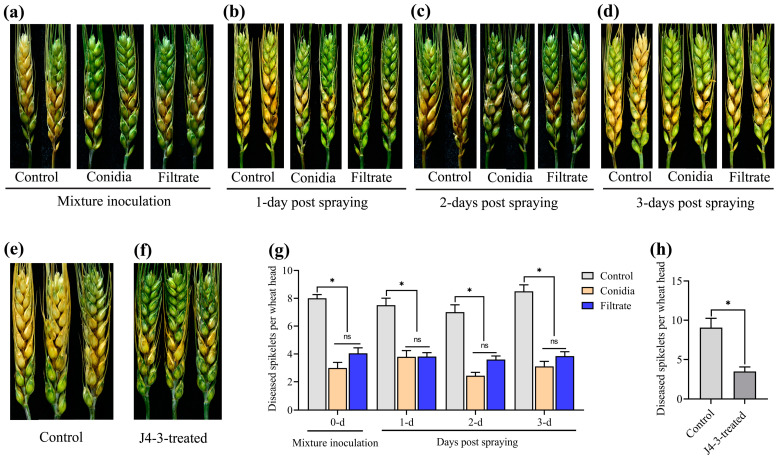
*E. layuense* enhances wheat resistance against FHB. (**a**–**d**) Spikes of wheat plants inoculated with *F. graminearum* under field conditions. (**a**) Mixture inoculation. (**b**–**d**) Inoculation of conidial suspensions of *F. graminearum*, one day, two, and three days after spraying of spore suspensions or culture filtrates of *E. layuense*, respectively. Sterile distilled water was used for untreated controls. (**e**,**f**) Spikes of wheat plants previously treated with spore suspensions of *E. layuense* or sterile distilled water before planting and inoculated with conidial suspensions of *F. graminearum* at the flowering stage. (**g**,**h**) Diseased spikelets per wheat head. (**g**) Mixture inoculation and inoculation of conidial suspensions of *F. graminearum* at one day, two, and three days post spraying of spore suspensions or culture filtrates of *E. layuense*, respectively. (**h**) Spikes of wheat plants previously treated with spore suspensions of *E. layuense* or sterile distilled water before planting. For each treatment, 15 wheat heads were inoculated, and images were taken at 15 dpi. Standard errors are represented by vertical bars. Significant differences at *p* ≤ 0.05 are denoted as *, and the non-significant difference is noted as ns according to Student’s *t*-tests or One-way ANOVA followed by Tukey’s HSD.

In the early stages, FHB symptoms first appeared on spikelets near the inoculated site, then spread to the adjacent spikelets. Significantly, at 15 dpi, the expansion of FHB symptoms on nontreated control spikelets was notably faster than on the *E. layuense*-treated plants (Figure 5a–d). The mixture inoculation of spore suspensions or culture filtrates of *E. layuense* with conidial suspensions of *F. graminearum* significantly reduced the diseased spikelet number (*p* ≤ 0.05). The reduction level was 62.5% and 49.4%, respectively. Applying conidial suspensions of *F. graminearum* at 1 dpi of spore suspensions or culture filtrates of *E. layuense* showed the significant biocontrol efficiency of FHB with reduction levels of 49.3% and 49.1%, respectively (*p* ≤ 0.05). In addition, at 2 dpi, the reduction levels were 65.2% and 48.5%, respectively (*p* ≤ 0.05). Moreover, applying conidial suspensions of *F. graminearum* at 3 dpi of spore suspensions or culture filtrates of *E. layuense*, the biocontrol efficacy of FHB was significant with a reduction level of 63.4% and 54.7%, respectively (*p* ≤ 0.05) (Figure 5g).

Furthermore, an experiment conducted in the field to investigate whether wheat seedlings treated with spore suspensions of *E. layuense* before planting could resist FHB at the anthesis stage revealed that the number of *E. layuense*-treated diseased spikelets per infected wheat head differed significantly (*p* ≤ 0.05) from the controls (Figure 5e,f). At 15 dpi, the inhibition level was 61.6% (Figure 5h).

### 3.6. E. layuense Strain J4-3 Promotes Wheat Growth under Greenhouse and Field Conditions

**Figure 6:**
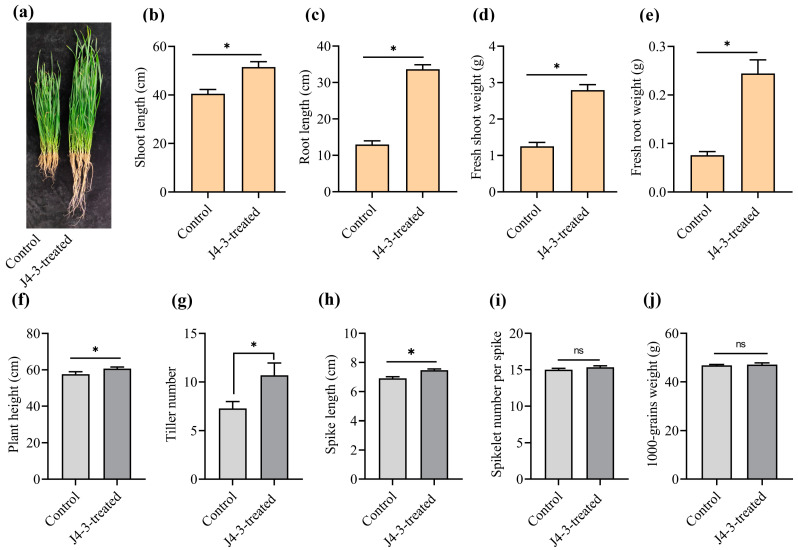
*E. layuense* strain J4-3 promotes wheat growth in greenhouse and field conditions. (**a**) Representative image of wheat plants treated with potato dextrose broth (control) and hyphal fragment suspensions of strain J4-3; seedlings were grown in a greenhouse for 45 days. (**b**) Shoot length (*t*-test, *p* ≤ 0.05) (*n* = 20). (**c**) Root length (*t*-test, *p* ≤ 0.05) (*n* = 20). (**d**) Fresh shoot weight (*t*-test, *p* ≤ 0.05) (*n* = 20). (**e**) Fresh root weight (*t*-test, *p* ≤ 0.05) (*n* = 20). The experiment was repeated three times with similar results. Under field conditions, plant growth parameters were measured, and data were analyzed. (**f**) Plant heights (*t*-test, *p* ≤ 0.05) (*n* = 20). (**g**) Tiller number (*t*-test, *p* ≤ 0.05) (*n* = 20). (**h**) Spike length (*t*-test, *p* ≤ 0.05) (*n* = 60). (**i**) Spikelet number per spike (*t*-test, *p* ≤ 0.05) (*n* = 60). (**j**) The weight of 1000 seeds of J4-3-treated and nontreated wheat plants in the field (*t*-test, *p* ≤ 0.05) (*n* = 5). The values represent the average ± s.e. Error bars indicate the standard error, and asterisks indicate a statistically significant difference according to the Student *t*-test at *p* ≤ 0.05. ns indicates no significance.

Under greenhouse conditions, *E. layuense*-treated plants grew more robustly compared to the control plants (Figure 6a). At the end of the experiment, the average shoot length (cm) of seedlings treated with strain J4-3 was 51.53 ± 2.17 cm, significantly greater than the untreated control plants (40.51 ± 1.77 cm) (*p* ≤ 0.05) (Figure 6b). The average root length (cm) of seedlings treated with strain J4-3 was 33.66 ± 1.18 cm, significantly greater than the nontreated control plants (12.98 ± 1.01 cm) (*p* ≤ 0.05) (Figure 6c). The average fresh shoot weight (g) of seedlings treated with strain J4-3 was 2.79 ± 0.15 g, significantly greater than the untreated control plants (1.25 ± 0.1 g) (*p* ≤ 0.05) (Figure 6d). The average fresh root weight (g) of seedlings treated with strain J4-3 was 0.24 ± 0.02 g, significantly greater than the nontreated control plants (0.07 ± 0.007 g) (*p* ≤ 0.05) (Figure 6e).

Under field conditions, wheat seedlings previously treated with spore suspensions of *E. layuense* or sterile distilled water revealed that the average height (cm) of plants treated with strain J4-3 was 60.68 ± 0.86 cm, significantly greater than the nontreated control plants (57.63 ± 1.35 cm) (*p* ≤ 0.05) (Figure 6f). The average tiller number of wheat plants treated with *E. layuense* was 10.68 ± 1.26, significantly greater than the control plants (7.26 ± 0.72 cm) (*p* ≤ 0.05) (Figure 6g). The average spike length (cm) of plants treated with *E. layuense* was 7.46 ± 0.08 cm, significantly greater than the nontreated control plants (6.91 ± 0.1 cm) (*p* ≤ 0.05) (Figure 6h). However, the average number of spikelets per spike for plants treated with *E. layuense* was 15.33 ± 0.21 cm, showing no significant difference compared to the nontreated control plants (15 ± 0.19 cm) (*p* ≤ 0.05) (Figure 6i). In addition, no significant differences were detected in the weight of 1000 seeds in treated plants (47.14 ± 0.29 g) (*p* ≤ 0.05) with controls (46.82 ± 0.15 g) (*p* ≤ 0.05) (Figure 6j and S1a,b).

## 4. Discussion

Plant diseases caused by fungal pathogens pose a serious threat to global food production. *F. graminearum*, a destructive fungal pathogen of wheat (*Triticum aestivum* L.), is well known for causing devastating wheat spike infections and damage to grain yield, resulting in significant yield losses [7]. FHB, caused by *F. graminearum*, has a significant impact on the production of wheat worldwide [52]. Biological control is a novel approach to controlling fungal diseases using microorganisms and microorganism-derived metabolites [21]. BCAs are beneficial microorganisms, including fungal endophytes, mycoviruses, and bacterial groups termed plant growth-promoting rhizobacteria (PGPR) [53]. Fungal endophytes are BCAs living inside host plants without inducing disease symptoms or producing external apparent injury [54,55]. In this study, strain J4-3 isolated from the stem of symptomless wheat was identified as *E. layuense* by morphological and molecular methods and significantly inhibited the growth of *F. graminearum*, a causal agent of FHB, and efficiently promoted wheat growth. 

The identification of fungal endophytes was historically based on morphological characteristics using culturing and microscopy features, such as conidia and the attributes of colonies [56]. Herein, strain J4-3 was initially identified using morphological characteristics. Interestingly, its morphological characteristics, including the color of the colonies, growth on PDA, shape, and size of conidia, were consistent with the *E. layuense* isolate H1 reported by Chen et al. [57]. However, it is difficult to confirm the species only by morphology due to the instability of morphological characteristics under altered conditions [58,59]. Thus, molecular analysis, a method that has been widely used to identify related species precisely, was performed using the ITS and *TUB* loci. Bian et al. [60], Chen et al. [57], and Koné et al. [20] conducted phylogenetic analyses using different primer sets, such as ITS and *TUB*, to accurately distinguish *Epicoccum* spp. Intriguingly, phylogenetic analyses provided evidence that the species obtained were related with 97–100% bootstrap support. Therefore, we postulated that these primer sets are good regions to amplify the identification of *Epicoccum* species.

In prior research, in vitro dual culture methods were used to assess the abilities of fungal endophytes as effective antagonists and producers of antimicrobial compounds against fungal pathogens [61,62]. According to Abaya et al. [1], mycelial plugs of fungal endophytes and pathogens were placed on PDA media using a dual confrontation test, and the formation of an inhibition zone was a criterion of antibiosis activity. Here, a dual culture test was initially performed to assess the in vitro biocontrol efficacy of *E. layuense* against various fungal pathogens, including *F. graminearum*, *B. cinerea*, *C. gloeosporioides*, and *S. sclerotiorum*. Notably, between pathogen colonies and *E. layuense*, inhibition of mycelial growth was observed with distinct inhibition zones. This result showed that strain J4-3 exhibited the same broad-spectrum antifungal effect previously demonstrated in other *Epicoccum* spp., such as occupying the growth space of fungal pathogens and slowing their hyphal growth [41,60].

Mycelial growth and conidial germination are important characteristics for the infection of fungal pathogens [63]. Intriguingly, the conidia of fungal pathogens play key roles in the infection of plants and disease spread in the field. When conidia are spread on plant parts, germination of conidia is an essential developmental step in the pathogenesis of disease [10]. Hence, inhibition of conidial germination is of considerable concern for disease management. Wheat coleoptiles are mostly feasible for tracking the processes of *F. graminearum* infection since the wheat coleoptile structure is simple, its infection test could be carried out in a growth chamber, and its growth is not limited by seasonal, geographical, or environmental factors [64]. In this research, the wheat coleoptile assay showed that Fusarium infections were strongly reduced in the *E. layuense*-treated seedlings compared to the untreated controls. According to these results, *E. layuense* could grow endophytically into wheat tissues, thereby inhibiting conidial germination of *F. graminearum* and, hence, inhibiting its growth and pathogenicity.

Fungal plant diseases decrease the yield and productivity of numerous economically important crops. FHB, a fungal disease caused by *F. graminearum*, is a devastating cereal crop disease that affects the yield and production of wheat [65]. The application of chemicals is frequently used as an intervention to quickly provide disease control [66]. Nevertheless, excessive use of chemicals causes problems, for instance, the increased resistance of pathogens and pesticide residues leading to pollution of the environment [67]. Therefore, using BCAs is crucial since they are economical, harmless, and eco-friendly [68]. In this study, we tested the possibility of controlling FHB using spore suspensions and culture filtrates of endophyte *E. layuense*. The mixture of treatments with conidial suspensions of *F. graminearum* at a 1:1 (*v*/*v*) ratio resulted in the significant biocontrol efficiency of FHB. Moreover, applying the treatments at 1, 2, and 3 days before the inoculation of conidial suspensions of *F. graminearum* significantly reduced the spread of FHB in wheat heads. Furthermore, wheat seedlings previously treated with spore suspensions of *E. layuense* before planting successfully restricted the spread of *F. graminearum* from the inoculation sites to nearby spikelets. Therefore, the obtained results showed that *E. layuense* considerably reduced the spread of FHB on wheat heads and could be used as a BCA against FHB in wheat.

Fungal endophytes have plant growth-promoting features that can enhance plant growth and production, thus serving as biofertilizers for agricultural applications [69]. Under greenhouse conditions, *E. nigrum* promoted potato plant growth [70]. In addition, it was found that rice seeds soaked with *E. nigrum* provided plant fitness and increased the number of tillers per plant compared to the untreated plants [20]. Furthermore, *E. nigrum* strain P16 isolated from the surface-disinfected leaves of healthy sugarcane induced the growth of a root system and biomass [71]. A noteworthy increase in seedling length and the fresh and dry weights of leguminous plants was observed in seedlings treated with *E. purpurascens* compared to the pathogen-treated seedlings [72]. Herein, an experiment conducted in a greenhouse under controlled conditions revealed a significant increase in wheat growth parameters, including shoot and root length and fresh shoot and root weights, in response to the treatment of hyphal fragment suspensions of *E. layuense* compared to the controls. These results suggested that strain J4-3 could be used as a biofertilizer for agricultural applications; however, additional experiments are warranted to confirm its application in plant growth-promoting efficacy further.

It was reported that all biocontrol agents effective under controlled conditions do not work successfully in the field due to the heterogeneity of conditions at the field level [63]. In this study, wheat seedlings treated with spore suspensions of *E. layuense* and grown in the field showed significant efficacy of growth parameters compared to the nontreated controls. Overall, our findings indicate the great potential of *E. layuense* strain J4-3 as a growth-promoting agent; however, more field experiments are essential to confirm its biocontrol potential for commercialization as a BCA.

## Data Availability

Data are contained within the article.

## Supplementary Materials

The following supporting information can be downloaded at: https://www.mdpi.com/article/10.3390/jof10010010/s1, Figure S1: The 1000 seeds of wheat seedlings treated with sterile distilled water (a), and spore suspensions of *E. layuense* strain J4-3 (b), before planting and grown until harvest under field conditions; Table S1: GenBank accession numbers of *Epicoccum* strains used for the phylogenetic analysis. The representative *E. layuense* strain J4-3 is in bold. References [39,47,73,74] are cited in Supplementary Materials.
