# Supplementary material for: The Wheat Endophyte Epicoccum layuense J4-3 Inhibits Fusarium graminearum and Enhances Plant Growth
Source: J Fungi (Basel). 2023 Dec 24;10(1):10. doi: 10.3390/jof10010010 (PMC10817605; doi:10.3390/jof10010010)
Supplement: Supplementary file 1 [file jof-10-00010-s001.zip › jof-2719438-supplementary.pdf]

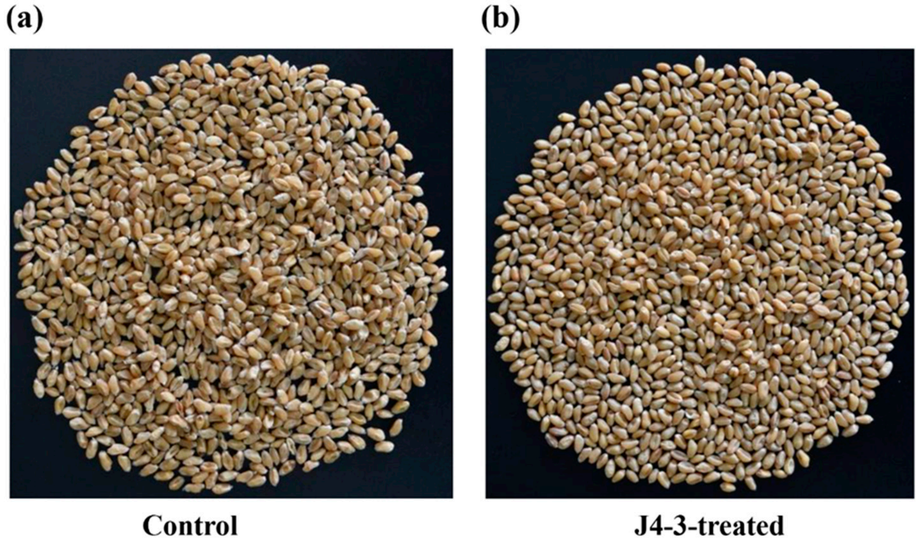

Figure S1. **(a,b)** The 1000 seeds of wheat seedlings treated with sterile water **(a)**, and spore suspensions of *E. layuense* strain J4-3 **(b)**, before planting and grown under field conditions until harvest.

Table S1. GenBank accession numbers of *Epicoccum* strains used for the phylogenetic analysis. The representative *E. layuense* strain J4-3 is in bold.

Species	Strain	Location	GenBank accession number		References
			ITS [1]	*TUB* [2]	
Epicoccum camelliae	LC 4858	China	KY742091	KY742333	Chen et al. [47]
E. camelliae	LC 4862	China	KY742092	KY742334	Chen et al. [47]
E. duchesneae	LC 5139	China	KY742095	KY742337	Chen et al. [47]
E. duchesneae	LC 8147	China	KY742096	KY742338	Chen et al. [47]
E. latusicollum	LC 5158	China	KY742101	KY742343	Chen et al. [47]
E. latusicollum	LC 4859	China	KY742102	KY742344	Chen et al. [47]
E. hordei	LC 8148	Australia	KY742097	KY742339	Chen et al. [47]
E. hordei	LC 8149	Australia	KY742098	KY742340	Chen et al. [47]
E. layuense	**J4-3**	**China**	**OR454088**	**OR536426**	**This study**
E. layuense	LC 8155	China	KY742107	KY742349	Chen et al. [47]
E. layuense	LC 8156	China	KY742108	KY742350	Chen et al. [47]
E. layuense	E 33	Portugal	MH643925	MH643935	Del Frari et al. [39]
E. sorghinum	CBS 179.80	Puerto Rico	FJ427067	FJ427173	Aveskamp et al. [73]
E. sorghinum	CBS 627.68	France	FJ427072	FJ427178	Aveskamp et al. [73]
E. pimprinum	CBS 246.60	India	FJ427049	FJ427159	Aveskamp et al. [73]
E. pimprinum	PD 77/1028	India	FJ427050	FJ427160	Aveskamp et al. [73]
Leptosphaeria doliolum	CBS 505.75	Netherlands	JF740205	JF740144	De Gruyter et al. [74]

[1] Internal transcribed spacer (ITS), and [2] beta-tubulin (*TUB*).
